# Associations between dietary vitamin K1 and K2 intake and incident dementia: findings from the Danish Diet, Cancer, and Health cohort

**DOI:** 10.1007/s00394-026-04081-w

**Published:** 2026-08-06

**Authors:** Negar Ghasemifard, Marc Sim, Pratik Pokharel, Chengfeng Li, Allan Linneberg, Anne Ahrendt Bjerregaard, Anja Olsen, Christina C. Dahm, Simone Radavelli-Bagatini, Simon M. Laws, Joshua R. Lewis, Jonathan M. Hodgson, Nicola P. Bondonno

**Affiliations:** 1 Nutrition and Health Innovation Research Institute, School of Medical and Health Sciences, Edith Cowan University, Perth, WA 6000 Australia; 2 Medical School, University of Western Australia, Crawley, WA Australia; 3 Royal Perth Hospital Research Foundation, Perth, WA Australia; 4 The Danish Cancer Institute, Copenhagen, Denmark; 5 Centre for Clinical Research and Prevention, Copenhagen University Hospital, Bispebjerg and Frederiksberg, Frederiksberg, Denmark; 6 Department of Clinical Medicine, The Faculty of Health and Medical Sciences, University of Copenhagen, Copenhagen, Denmark; 7 Department of Public Health, Aarhus University, Aarhus, Denmark; 8 Centre for Precision Health, Edith Cowan University, Joondalup, WA Australia; 9 Collaborative Genomics and Translation Group, School of Medical and Health Sciences, Edith Cowan University, Joondalup, WA Australia

**Keywords:** Diet, Epidemiology, Vitamin K, Dementia

## Abstract

**Purpose:**

Vitamin K is hypothesised to support vascular integrity and neuronal function through vitamin K-dependent proteins, yet epidemiological evidence for its role in dementia, particularly distinguishing vitamin K1 and K2, is limited. This aetiological study examined associations between dietary vitamin K1 and K2 with incident dementia in a large Danish cohort.

**Methods:**

We included 54,968 dementia-free participants (47.5% male, aged 50–65 years) from the Danish Diet, Cancer, and Health cohort (1993–1997) with dietary vitamin K1 (µg/day), and K2 (µg phylloquinone equivalent/day) intakes estimated from a 192-item food frequency questionnaire. Incident and early-onset dementia were identified through national registries. Cox proportional hazard models with restricted cubic splines estimated hazard ratios (HR) across quintiles of intake, adjusting for demographic, lifestyle, dietary factors, and prevalent comorbidities.

**Results:**

Over a maximum of 28 years of follow-up (median [IQR]: 25.5 [16.1–26.5] years), 4802 (8.7%) participants developed dementia. Vitamin K1 intake showed an inverse association with dementia, with a 14% lower rate in quintile (Q) 5 versus Q1 (HR _Q5vsQ1_ [95%CI]: 0.86 [0.78–0.93]). The association was stronger for early-onset dementia (< 65 years). In contrast, vitamin K2 intake was associated with higher dementia risk, whereby those with the highest intakes had 14% higher rate (HR_Q5vsQ1_ [95%CI]: 1.14 [1.04–1.24]).

**Conclusion:**

Higher vitamin K1 intake was associated with lower dementia risk, while higher K2 intake was associated with higher risk. These associations may be confounded by dietary patterns and food sources associated with each vitamin form and warrant further investigations. Nevertheless, greater intake of vitamin K1-rich vegetables (e.g. spinach, cabbages, broccoli, etc.) as part of broader dietary patterns may be relevant to cognitive health.

**Supplementary Information:**

The online version contains supplementary material available at https://doi.org/10.1007/s00394-026-04081-w.

## Introduction

The global population is aging rapidly, with the prevalence of chronic diseases such as dementia consistently increasing [1]. Dementia is a major public health concern with one new case diagnosed every three seconds [1, 2]. By 2030, cases are projected to reach 74.7 million, with healthcare costs of US$2 trillion each year [2]. The complex pathology of dementia combined with the limited efficacy of current pharmacological treatments necessitate multifaceted prevention and treatment approaches [3, 4]. The 2024 Lancet commission identified 14 modifiable risk factors that if addressed, could prevent up to 50% of dementia cases globally [5]. Although diet is not explicitly listed, it is linked to at least five of the listed cardiometabolic risk factors (diabetes, obesity, high cholesterol, hypertension, and alcohol intake); suggesting the potential role of nutrition in dementia prevention [5, 6].

Vitamin K is best known for its role in coagulation, with some evidence also pointing to benefits for bone and cardiovascular health [7]. Emerging research suggests that vitamin K may also be a contributor to cognitive health [8]. Such benefits may arise from the activation of vitamin K-dependent proteins (VKDPs), including pro-thrombotic proteins (coagulation factors II, VII, IX, and X) and anti-thrombotic factors (Protein S and Protein C) that influence dementia risk through their effects on thrombosis and cerebrovascular integrity [8, 9]. Other VKDPs such as osteocalcin, growth arrest-specific protein 6 (Gas6), and matrix Gla protein (MGP), may reduce neuroinflammation and Aβ plaque accumulation, suppress the formation of reactive oxygen species, and/or inhibit vascular calcification to maintain microvascular elasticity [4, 8, 10]. Warfarin, a vitamin K antagonist, is also associated with greater dementia risk, compared with direct oral anticoagulants (DOACs) [11]. Vitamin K exists in two main forms: vitamin K1 (phylloquinone, PK) is abundant in plant-based foods, particularly green leafy vegetables and plant oils, while vitamin K2 (menaquinone, MK) is primarily found in animal-derived products and fermented foods such as meat, cheese and yoghurt [12]. These MKs have side chains of varying length (MK-4 to MK-13), which influence their absorption, metabolism, and bioactivity [8].

Cross-sectional studies have reported lower dietary vitamin K1 intake in patients with early-stage Alzheimer’s disease compared to cognitively healthy individuals [13]. They have also found associations between higher dietary intake or serum concentrations of vitamin K1 and better cognitive performance [14–16]. Biomarkers of vitamin K insufficiency, such as undercarboxylated osteocalcin, have also been linked to greater odds of cognitive impairment [17]. However, findings from prospective studies are limited and inconsistent [18, 19], and most of this evidence has focused on vitamin K1 and total vitamin K. Vitamin K2 has been rarely examined despite having distinct dietary sources and/or biological functions that could differentially influence brain health [20]. This is likely attributed to the lack of comprehensive vitamin K2 food databases [7] which have only recently become available [12, 21].

The primary aim of this study was to examine the associations between dietary intakes of vitamin K1 and K2 (separately) and the incidence of dementia in the Danish Diet, Cancer, and Health (DDCH) cohort. Secondary aim was examining the associations between dietary intakes of vitamin K1 and K2 (separately) and cases of early-onset dementia only (defined as a dementia diagnosis before the age of 65 years) which is thought to be more strongly influenced by lifestyle factors than late-onset dementia [22].

## Materials & methods

### Study population

From December 1993 to May 1997, a total of 160,725 men and women between the ages of 50 to 65 years, residing in the greater Copenhagen and Aarhus areas, were invited to participate in the DDCH study [23]. A final cohort of 57,053 individuals with no prior cancer diagnosis were enrolled. Participants were recruited using Denmark’s unique civil registration numbers, enabling individual-level linkage to several nationwide registries. These included the Civil Registration System [24], the Danish National Patient Register (DNPR) [25], the Danish National Prescription Registry [26], the National Death Registry [27], the Register for Selected Chronic Diseases (RUKS) [28], and the Education Registry [29]. These registries provided detailed information on demographic data, diagnoses, prescriptions, and other health-related factors. Since complete data in the RUKS registry was only available from 1st January 1995, this date was designated as the baseline for all participants enrolled prior to 1995 (*n* = 4987).

For the present study, participants were excluded if they died or immigrated before 1st January 1995, or had prevalent cancer diagnosed with delayed registration (*n* = 596), or if they had prevalent dementia at baseline (*n* = 16) –defined as a recorded diagnosis of dementia in the DNPR (which keeps records since 1977; International Classification of Diseases, Revision 8 [ICD-8]: 29009–29011, 29018, 29019, 29309, and 29319; ICD-10: F00–F02, F039, and G30), or a documented record of dementia in RUKS prior to baseline (see detailed definition below in “Outcome” section). Moreover, 255 individuals were excluded due to the use of vitamin K antagonists (e.g., warfarin) at baseline. Additional exclusions included those with missing dietary (*n* = 95) or covariate (*n* = 1123) data. After applying all exclusion criteria, a total of 54,968 participants remained eligible for inclusion in the final analysis (Supplementary Fig. 1). All participants provided written informed consent. The DDCH study was approved by the relevant regional scientific ethics committees and the Danish Data Protection Agency.

### Exposures

Dietary intakes of vitamin K1 and vitamin K2 (MK-4 to MK-10) were the primary exposures in this study. These data were collected using a validated semi-quantitative food frequency questionnaire (FFQ) [30], which was emailed to participants prior to their first study visit. The FFQ assessed habitual dietary intake over the previous 12 months and included 192 food and beverage items. For each item, participants reported their usual frequency of consumption using a 12-category scale, ranging from “never” to “eight or more times per day” [30–32]. Habitual intake of each food and beverage item was calculated for each participant using the program FoodCalc [33], which used standardised recipes and portion sizes specifically developed for this FFQ. Dietary intake of vitamin K1 and K2 was estimated by multiplying the amount of food or beverage consumed (g/day) by the corresponding vitamin K1 (µg/g) or K2 (µg PK equivalent [PKeq]/g) content, as reported in available vitamin K databases.

Estimates of vitamin K1 content (µg/g) for each food item were primarily sourced from the recent analysis of 88 composite food samples conducted by Jensen et al. [21]. When a value was not available from this database, data was subsequentially retrieved from a hierarchy of databases in the following order: the Danish Frida Food Data database [34], the Dutch food composition database [35], the UK McCance and Widdowson’s Composition of Foods Integrated Dataset [36], the USDA National Nutrient Database [37], and data from Australia [12]. If no value could be assigned for a food item across any of these sources, a value of 0 µg/g was assigned. This stepwise approach was applied to prioritise data originating from the country of interest, followed by neighbouring countries, then the region, and finally, other regions when necessary.

Likewise, the estimates of vitamin K2 (µg PKeq/g) content (including MK-4 through MK-10) followed the same hierarchical approach from one database to the other. Primarily values were taken from the recent Jensen et al. [21], and additional values supplemented from the same alternative databases used for vitamin K1 as well as from other sources such as Schurgers et al. [38], and Manoury et al. [39]. Due to the differences in molecular weights across menaquinone isoforms, individual MKs were converted into their PK equivalents (PKeq) [21] to standardise the various forms as a common unit to allow aggregation of total vitamin K2 intake. Total dietary vitamin K2 intake was then calculated by summing the individual menaquinone contributions, expressed as PK equivalents, across all food and beverage items reported in the FFQ.

## Outcomes

The primary outcome in this study was incident dementia. Briefly, dementia was identified through the RUKS registry based on either a validated hospital diagnosis recorded in The Danish National Patient Registry [ICD-10 codes: F00, F01, F02, F03, G30, G31.0B, G31.8, G31.8E, G31.9] [25], or the prescription of a dementia-related medication [Anatomical Therapeutic Chemical (ATC) code: N06D] recorded in the Danish National Prescription Registry [26] from baseline until 22^nd^ September 2022. Further details on the algorithm used in the RUKS registry is described elsewhere [28]. The secondary outcome was early-onset dementia, defined as a dementia diagnosis before 65 years of age.

## Covariates

Upon enrolment, participants completed a self-administered questionnaire providing information about various demographic and lifestyle factors (including age, sex, smoking habits, diet, and leisure-time physical activity during both summer and winter). At their study centre visit, height (m) and weight (kg) were measured by trained nurses using standardised equipment, and body mass index (BMI) was subsequently calculated (kg/m^2^). Data pertaining to participants’ education level and living situation (being single or living with a partner) were obtained from Statistics Denmark. More details are presented in Supplementary Table 1.

Information on prevalent health conditions was obtained from DNPR and the RUKS registry, described in Supplementary Table 1. Briefly, pre-existing prevalent chronic kidney disease (CKD), and CVD (including ischemic heart disease, ischemic stroke, haemorrhagic stroke, peripheral artery disease) were defined as a record of disease identified using relevant ICD-8 and ICD‐10 codes. Prevalent diabetes was defined as a record of either type 1 or type 2 diabetes in the RUKS registry prior to baseline. Use of vitamin K antagonists (VKAs), antihypertensive medications, and cholesterol-lowering medications (statins) were identified from the Prescription Registry using the ATC codes described in Supplementary Table 1.

## Statistical analyses

All statistical analyses were conducted using R Studio statistical software (version 2024.12.1; R Foundation for Statistical Computing). Descriptive statistics were used to summarise baseline characteristics for the whole cohort as well as by low (quintile 1) and high (quintile 5) intakes of vitamin K1 and K2. Cox proportional hazard models were utilised to analyse the association between dietary intakes of vitamin K1 and vitamin K2 (separately) with incident dementia. Follow-up time was calculated from 1^st^ of January 1995 (for participants enrolled before 1995) or from the enrolment date, whichever came later, until the date of dementia diagnosis, death, emigration, initiation of VKA treatment, or the end of follow-up (22^nd^ September 2022), whichever occurred first. To allow for non-linear relationships between exposures and outcomes, continuous exposure variables were fitted as restricted cubic splines (with 4 knots placed at the 5^th^, 35^th^, 65^th^, and 95^th^percentiles). Hazard ratio (HR) estimates were calculated for the median intake of each quintile, with the median of quintile 1 (lowest intake) used as the reference point. Results were displayed graphically for the outcome with 95% confidence bands. For visual simplicity, the spline plots had x-axis values restricted to intakes within 3 standard deviations of the mean for each exposure. The proportional hazards assumption was assessed by visual inspection of the scaled Schoenfeld residual plots and formal tests which showed no evidence of meaningful violation for both vitamin K1 (*p* value = 0.08) and K2 (*p* value = 0.47). To examine the associations between dietary intakes of vitamin K1 and vitamin K2 (separately) and early-onset dementia, participants were censored at the time they turned 65 years.

Covariates were chosen *a-priori* based on knowledge of potential confounders of the relationship between vitamin K intake and dementia. Three models of adjustment were used: Model 1 adjusted for sex and age, Model 2 included Model 1 plus BMI, physical activity, education level, smoking status, smoking pack per year, living situation, and alcohol intake. Model 3 included all covariates in Model 2 plus dietary covariates tailored to the vitamin K form examined: (a) when plant-based vitamin K (K1) was the exposure of interest, the model was further adjusted for intakes of red meat, poultry, processed meat, fish, dairy, refined grains, tea, coffee, and sugar-sweetened soft drinks; (b) when animal-based vitamin K (K2) was the exposure of interest, the model was further adjusted for intakes of vegetables, fruits, wholegrains, refined grains, tea, coffee, and sugar-sweetened soft drinks. Model 3 was selected as the main model as it accounts for additional residual confounding from dietary factors which can influence the relationship between vitamin K and dementia. This approach (adjusting simultaneously for individual food components rather than total energy intake) is consistent with the “all-components model” described by Tomova et al. [40], which has been shown to be the most conservative method and provides less biased estimate of total causal effect compared to standard energy adjustment approaches [40].

Additional analyses included: (i) examining associations between intakes of individual vitamin K2 vitamers that contributed > 10% to total K2 intakes (MK-4 and MK-9) and dementia incidence; (ii) generating cumulative incidence plots of dementia by quintiles of vitamin K1 and vitamin K2 using the Aalen-Johansen estimator, accounting for death as a competing risk, as previous research in this cohort has shown associations between the exposure of this study (vitamin K) and mortality [41]; and (iii) assessing effect modification of sex, BMI, smoking status (never vs. ever), and prevalent diabetes by running likelihood ratio tests to test for interactions between vitamin K exposures (K1 and K2) and these variables.

To assess the robustness of our findings, we performed several sensitivity analyses: (i) to examine the potential influence of energy intake on the vitamin K-dementia association, total energy intake was added as an additional covariate to Model 2. This was applied to Model 2 because additional energy adjustment in Model 3, which already included multiple absolute food-group intake may introduce multi-collinearity and reduce interpretability [40]; (ii) to account for baseline comorbidities, history of diabetes, CVD, and CKD, as well as the use of antihypertensive and cholesterol-lowering medications were added to Model 3; (iii) to minimise bias from probable inaccurate dietary reporting, participants with potentially implausible energy intakes (defined as < 800 or > 4200 kcal/day for men and < 500 or > 3500 kcal/day for women [42]) were excluded (*n* = 972); (iv) to reduce the potential for reverse causation, participants diagnosed with dementia within 5 years from baseline were excluded from the analyses (excluded participants *n* = 2843); (v) the follow-up period was restricted to 10 years to assess if associations were stronger when the dietary assessment was more temporally proximate to the outcome; finally (vi) given that processed meat consumption is linked to less healthy dietary patterns and has been associated with higher risk of chronic diseases [43, 44], processed meat intake was additionally included in the fully adjusted vitamin K2 model (Model 3b) to examine whether it influences the association between vitamin K2 intake and dementia.

## Results

This population of 54,968 Danish citizens (52.5% female) with a median [IQR] age of 56.0 [52.0–60.0] years at enrolment, had a median [IQR] follow-up time of 25.5 [16.1–26.5] years. During a maximum of 28 years of follow-up, 4802 individuals (8.7%) were diagnosed with dementia, with a mean ± SD age at diagnosis of 77.7 ± 7.7 years. Food group contributions to total vitamin K1 and vitamin K2 intake are presented in Supplementary Fig. 2. The median [IQR] intake of vitamin K1 was 88.1 [65.5-115.3] µg/day. The top dietary sources contributing more than 10% to vitamin K1 intake were potatoes (26%), cabbages (22%), and green leafy vegetables (16%), collectively accounting for ~ 64% of total intake (Supplementary Fig. 2a). The median [IQR] intake of vitamin K2 was 47.8 [37.3–60.3] µg PKeq/day. On average, MK-4 (primarily from red meat, eggs, and poultry) and MK-9 (mainly from dairy products) contributed to 71.5% and 15.8% of total vitamin K2 intake, respectively, representing the top two contributors of total MK intake. The five leading sources of vitamin K2 included fatty dairy products (20%), red meat (19%), eggs (18%), lean dairy products (17%), and poultry (17%), totalling approximately 90% of overall vitamin K2 intake (Supplementary Fig. 2b).

## Baseline characteristics

Baseline characteristics of the study population overall and by lowest (Q1) and highest (Q5) quintiles of vitamin K1 and K2 intakes are presented in Table 1. Compared to participants with the lowest vitamin K1 intakes, a higher proportion of those with the highest intakes were male and non-smokers, they tended to be more physically active and more likely to have higher educational attainment and live with a partner. They were also less likely to be hypertensive or present with CVD, but more likely to have diabetes. They also reported higher intakes of total energy, vegetables, fruits, whole grains, red meat, fish, dairy, tea, and alcohol. Similar patterns were observed for participants with the highest, compared to those with the lowest intakes of vitamin K2, except that they tended to have smoked more and were less likely to present with hypercholesterolemia. The discrepancy in intakes of plant- and animal-based foods was lower and higher, respectively.

**Table 1:** Baseline characteristics of study population

	Total population *n* = 54,968	Total vitamin K1 intake quintiles		Total vitamin K2 intake quintiles	
		Quintile 1 *n* = 10,994	Quintile 5 *n* = 10,993	Quintile 1 *n* = 10,994	Quintile 5 *n* = 10,993
Total vitamin K1 intake, µg/day	88.1 [65.5–115.3]	48.8 [40.6–55.0]	146.2 [132.5–168.4]	69.9 [50.7–93.9]	108.3 [84.0–137.6]
Total vitamin K2 intake, µg PKeq/day	47.8 [37.3–60.3]	39.0 [30.5–48.9]	57.0 [45.0–71.0]	29.1 [25.0–32.4]	74.4 [68.3–84.5]
*Sociodemographic*					
Sex, male	26,088 (47.5)	4607 (41.9)	5676 (51.6)	3144 (28.6)	6989 (63.6)
Age, year	56.0 [52.0–60.0]	56.0 [52.0–60.0]	55.0 [52.0–60.0]	56.0 [52.0–60.0]	56.0 [52.0–60.0]
BMI, kg/m^2^	25.5 [23.3–28.2]	26.0 [23.5–28.9]	25.1 [23.0–27.6]	25.1 [22.8–27.8]	26.1 [23.8–28.8]
MET score	56.5 [37.0–85.0]	49.1 [31.0–76.5]	63.5 [42.0–92.5]	52.5 [34.0–77.7]	62.0 [39.5–93.0]
Smoking status					
Current	19,865 (36.1)	5046 (45.9)	3278 (29.8)	4047 (36.8)	4130 (37.6)
Former	15,696 (28.6)	2550 (23.2)	3595 (32.7)	2823 (25.7)	3399 (30.9)
Never	19,407 (35.3)	3398 (30.9)	4120 (37.5)	4124 (37.5)	3464 (31.5)
Cigarette packyears	9.3 [0.0–26.3]	16.0 [0.0–30.5]	6.0 [0.0–22.8]	7.3 [0.0–24.5]	13.0 [0.0 -29.6]
Education					
Short	10,107 (18.4)	2747 (25.0)	1394 (12.7)	2232 (20.3)	2139 (19.5)
Medium	30,003 (54.6)	6591 (60.0)	5309 (48.3)	6176 (56.2)	5845 (53.2)
Higher	14,858 (27.0)	1656 (15.1)	4290 (39.0)	2586 (23.5)	3009 (27.4)
Living situation, single	14,349 (26.2)	3717 (33.9)	2682 (24.5)	3475 (31.7)	2977 (27.1)
*Comorbidities*					
CVD	2796 (5.1)	660 (6.0)	465 (4.2)	585 (5.3)	612 (5.6)
Diabetes	756 (1.4)	117 (1.1)	228 (2.1)	85 (0.8)	277 (2.5)
CKD	131 (0.2)	25 (0.2)	22 (0.2)	19 (0.2)	34 (0.3)
*Medication use*					
Antihypertensive	2235 (4.1)	521 (4.7)	386 (3.5)	444 (4.0)	438 (4.0)
Statin	588 (1.1)	99 (0.9)	110 (1.0)	152 (1.4)	89 (0.8)
*Dietary characteristics*					
Energy, Kcal/day	2270.8 [1877.9–2718.9]	1851.1 [1545.5–2214.0]	2689.1 [2285.7–3176.1]	1748.7 [1480.4–2048.9]	2924.8 [2521.3–3380.3]
Vegetable intake, g/day	161.3 [104.4–230.8]	79.3 [53.9–110.9]	274.3 [212.8–347.2]	123.8 [74.2–190.6]	206.2 [143.3–283.4]
Fruit intake, g/day	171.5 [94.7–281.5]	112.4 [52.0–190.1]	243.6 [148.2–383.3]	145.3 [72.6–250.8]	201.1 [118.4–328.1]
Refined grain intake, g/day	45.8 [29.3–72.3]	41.8 [24.9–69.0]	49.7 [32.4–74.6]	35.8 [22.0–53.8]	58.4 [38.5–93.5]
Whole grain intake, g/day	39.0 [25.0- 56.4]	28.7 [21.6–43.9]	47.6 [31.9–65.4]	29.5 [21.6–46.0]	46.5 [31.3–64.7]
Red meat intake, g/day	78.4 [56.6–107.1]	67.1 [49.0–90.1]	86.8 [59.7–120.9]	55.6 [40.8–72.1]	111.8 [80.5–149.5]
Poultry intake, g/day	17.9 [10.2–27.5]	12.3 [6.7–19.9]	23.7 [14.0–36.0]	9.8 [5.5–15.3]	31.1 [19.6–49.0]
Processed meat intake, g/day	24.6 [14.2–40.1]	23.0 [13.4–37.2]	24.8 [13.0–42.2]	15.8 [9.0–25.2]	37.3 [22.0–58.4]
Total fish intake, g/day	38.2 [25.4–55.3]	27.9 [17.9–40.0]	50.1 [34.2–71.2]	27.3 [17.8–39.2]	53.2 [36.1–74.7]
Dairy intake, g/day	295.9 [155.6–561.2]	251.2 [110.0–529.5]	348.4 [207.9–609.2]	226.2 [82.3–405.0]	412.8 [257.4–687.0]
Sugar/confectionary intake, g/day	46.4 [26.4–77.9]	38.4 [20.8–68.4]	52.0 [30.3–86.5]	38.1 [21.0–65.8]	56.6 [32.4–96.3]
Tea intake, ml/day	85.7 [3.3–500.0]	16.4 [3.3–200.0]	200.0 [16.4–500.0]	85.7 [3.3–500.0]	85.7 [6.6–500.0]
Coffee intake, ml/day	900.0 [500.0–1300.0]	900.0 [500.0–1300.0]	900.0 [500.0–900.0]	900.0 [500.0–900.0]	900.0 [500.0–1300.0]
Sugar-sweetened soft drink intake, ml/day	8.0 [1.4–29.1]	8.0 [1.4–29.1]	7.1 [1.4–23.6]	5.7 [1.4–17.8]	16.7 [2.4–30.0]
Vegetable oil intake, ml/day	4.7 [1.2–8.9]	1.3 [0.6–4.2]	8.5 [2.7–13.3]	2.0 [0.7–6.0]	5.3 [1.6–11.8]
Alcohol intake, g/day	12.9 [5.9–31.0]	11.3 [3.1–31.0]	14.1 [6.7–31.3]	10.4 [2.9–23.0]	15.4 [7.0–33.8]
Butter intake, g/day	9.2 [1.0–20.1]	7.1 [0.9–16.8]	11.1 [0.9–24.2]	6.5 [0.6–16.1]	12.6 [0.9–25.2]

Data expressed as median [IQR] for non-normally distributed continuous variables and n (%) for categorical variables.

Phylloquinone equivalent (PKeq), Body Mass Index (BMI), Metabolic Equivalent (MET), Cardiovascular Disease (CVD), Chronic Kidney Disease (CKD).

## Vitamin K intakes and incident dementia

**Fig. 1:**
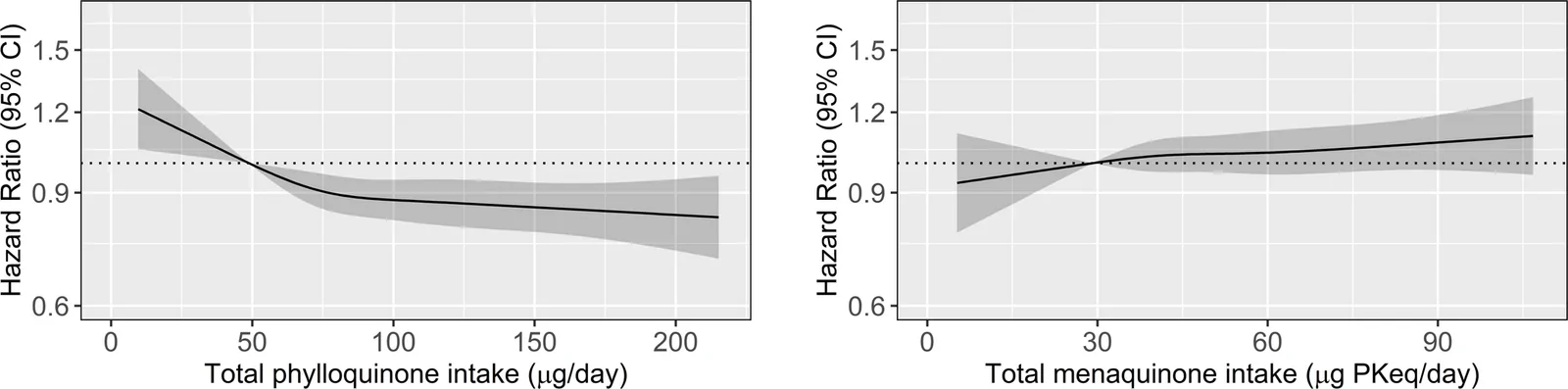
Hazard ratios (HRs) from Cox proportional hazards model with restricted cubic spline curves describing the association between (**a)** vitamin K1 (phylloquinone, [µg/day]) and (**b)** vitamin K2 (menaquinone, µg Phylloquinone equivalent [PKeq]/day) intake and incident dementia over 28 years. HRs for (a) are based on model adjusted for sex, age, body mass index, physical activity, education, smoking status, smoking pack per year, living situation, alcohol intake, intakes of red meat, poultry, processed meat, fish, dairy, refined grains, tea, coffee and sugar-sweetened soft drink (model 3a) and are comparing the specific level of vitamin K1 intake (horizontal axis) to the median intake for participants in the lowest-intake quintile (48.8 µg/day). HRs for (b) are based on model adjusted for sex, age, body mass index, physical activity, education, smoking status, smoking pack per year, living situation, intake of vegetables, fruits, wholegrains, refined grains, tea, coffee and sugar-sweetened soft drink (model 3b) and are comparing the specific level of vitamin K2 intake (horizontal axis) to the median intake for participants in the lowest-intake quintile (29.1 µg PKeq/day). Solid lines are the estimated HR, and shaded areas represent the 95% confidence intervals

Intakes of vitamin K1 were inversely associated with dementia incidence, whereby higher intakes were associated with lower hazard ratios. Although the test for non-linearity did not reach statistical significance (*p* for non-linearity = 0.0603), the spline shape visually suggested a possible non-linear association (Fig. 1a). Compared to participants in the lowest quintile intakes, those in the highest quintile had 14% lower rate of dementia (HR_Q5vsQ1_ [95%CI]: 0.86 [0.78–0.93]; Model 3a, Table 2), with risk reduction possibly plateauing from Q3 at intakes of approximately 90 µg/day (HR_Q3vsQ1_ [95%CI]: 0.88 [0.83–0.95]; Model 3a, Table 2). In contrast, higher intakes of vitamin K2 were associated with higher rates of incident dementia (Fig. 1b). Compared to individuals with the lowest intakes, those with the highest intakes had 14% higher relative hazards for incident dementia (HR_Q5vsQ1_ [95%CI]: 1.14 [1.04–1.24]; Model 3b, Table 2).

**Table 2:** Hazard ratios for incident dementia by quintiles of vitamin K intake

	Vitamin K intake quintiles				
	Quintile 1 *n* = 10,994	Quintile 2 *n* = 10,994	Quintile 3 *n* = 10,994	Quintile 4 *n* = 10,993	Quintile 5 *n* = 10,993
Vitamin K1					
Intake, µg/day ^a^	48.8 [40.6–55.0]	70.2 [65.5–74.8]	88.1 [83.6–93.0]	108.8 [103.0–115.3]	146.2 [132.5–168.4]
Number of events (%)	1011 (9.2)	957 (8.7)	942 (8.6)	977 (8.9)	915 (8.3)
HR (95%CI)					
Model 1	Ref.	**0.90 (0.85–0.95)**	**0.86 (0.81–0.92)**	**0.86 (0.80–0.92)**	**0.85 (0.79–0.92)**
Model 2	Ref.	**0.93 (0.88–0.98)**	**0.90 (0.85–0.96)**	**0.90 (0.84–0.97)**	**0.90 (0.83–0.97)**
Model 3a	Ref.	**0.92 (0.86–0.97)**	**0.88 (0.83–0.95)**	**0.87 (0.81–0.94)**	**0.86 (0.78–0.93)**
Vitamin K2					
Intake, µg PKeq/day ^a^	29.1 [25.0–32.4]	39.5 [37.3–41.6]	47.8 [45.8–50.0]	57.3 [54.6–60.3]	74.4 [68.3–84.5]
Number of events (%)	943 (8.6)	973 (8.9)	966 (8.8)	941 (8.6)	979 (8.9)
HR (95%CI)					
Model 1	Ref.	1.01 (0.95–1.06)	1.02 (0.96–1.08)	1.03 (0.96–1.11)	1.07 (0.99–1.16)
Model 2	Ref.	1.03 (0.97–1.08)	1.04 (0.97–1.10)	1.04 (0.97–1.12)	1.07 (0.99–1.16)
Model 3b	Ref.	**1.05 (1.00-1.12)**	**1.08 (1.01–1.16)**	**1.10 (1.02–1.19)**	**1.14 (1.04–1.24)**

Hazard ratios (95% confidence intervals) for dementia incidence over 28 years of follow-up, obtained from restricted cubic splines based on Cox proportional hazards models, comparing the median intake in quintiles 2–5, to the median intake in quintile 1. Bolded indicates *p* < 0.05 compared to Q1. Model 1 adjusted for sex and age; model 2 adjusted for sex, age, body mass index, physical activity, education, smoking status, smoking pack per year, living situation, and alcohol intake; model 3a adjusted for all covariates in model 2 plus intakes of red meat, poultry, processed meat, fish, dairy, refined grains, tea, coffee and sugar-sweetened soft drink when vitamin K1 is the exposure of interest; model 3b adjusted for all covariates in model 2 plus intake of vegetables, fruits, wholegrains, refined grains, tea, coffee and sugar-sweetened soft drink when vitamin K2 is the exposure of interest.

Phylloquinone equivalent (PKeq), Hazard ratio (HR), 95% confidence interval (CI).

^a^ Median [interquartile range]

When examining the association with cases of early-onset dementia, 695 participants who were already 65 years old at baseline were excluded. Among 54,273 participants in this analysis, 186 (0.34%) individuals were diagnosed with dementia before the age of 65 years over a maximum 15 years of follow-up. Higher vitamin K1 intakes were inversely associated with early-onset dementia, with estimates suggesting a plateau at intakes of approximately 90 µg/day. Compared to individuals in the lowest quintile of intake, those in the highest quintile had a 38% lower rate of early-onset dementia in the fully adjusted analysis (HR_Q5vsQ1_ [95%CI]: 0.62 [0.39–0.96]; Model 3a, Table 3), with no further risk reduction beyond Q3. No associations were observed between vitamin K2 intake and early-onset dementia.

**Table 3:** Hazard ratios for early-onset incident dementia by quintiles of vitamin K intake

	Vitamin K intake quintiles				
	Quintile 1 *n* = 10,855	Quintile 2 *n* = 10,855	Quintile 3 *n* = 10,855	Quintile 4 *n* = 10,854	Quintile 5 *n* = 10,854
Vitamin K1					
Intake, µg/day ^a^	48.8 [40.6–55.0]	70.3 [65.5–74.8]	88.1 [83.6–93.0]	108.8 [103.0–115.3]	146.1 [132.5–168.4]
Number of events (%)	61	28	35	29	33
HR (95%CI)					
Model 1	Ref.	**0.60 (0.48–0.74)**	**0.52 (0.40–0.66)**	**0.53 (0.38–0.74)**	**0.55 (0.37–0.80)**
Model 2	Ref.	**0.65 (0.53–0.81)**	**0.58 (0.45–0.75)**	**0.60 (0.43–0.85)**	**0.62 (0.42–0.92)**
Model 3a	Ref.	**0.69 (0.55–0.87)**	**0.62 (0.47–0.82)**	**0.62 (0.42–0.91)**	**0.62 (0.39–0.96)**
Vitamin K2					
Intake, µg PKeq/day ^a^	29.1 [25.0–32.4]	39.6 [37.3–41.6]	47.8 [45.8–50.0]	57.3 [54.6–60.3]	74.3 [68.3–84.5]
Number of events	40	43	31	37	35
HR (95%CI)					
Model 1	Ref.	1.03 (0.77–1.37)	0.87 (0.63–1.20)	0.69 (0.47–0.99)	0.66 (0.44-1.00)
Model 2	Ref.	1.09 (0.82–1.45)	0.94 (0.68–1.31)	0.75 (0.52–1.09)	0.71 (0.47–1.08)
Model 3b	Ref.	1.17 (0.87–1.56)	1.05 (0.74–1.48)	0.85 (0.57–1.27)	0.82 (0.52–1.30)

Hazard ratios (95% confidence intervals) for early-onset dementia incident over 15 years of follow-up (*n* = 54,273). Hazard ratios obtained from restricted cubic splines based on Cox proportional hazards models, comparing the median intake in quintiles 2–5, to the median intake in quintile 1. Bolded indicates *p* < 0.05 compared to Q1. Model 1 adjusted for sex and age; model 2 adjusted for sex, age, body mass index, physical activity, education, smoking status, smoking pack per year, living situation, and alcohol intake; model 3a adjusted for all covariates in model 2 plus intakes of red meat, poultry, processed meat, fish, dairy, refined grains, tea, coffee and sugar-sweetened soft drink; model 3b adjusted for all covariates in model 2 plus intake of vegetables, fruits, wholegrains, refined grains, tea, coffee and sugar-sweetened soft drink.

Phylloquinone equivalent (PKeq), Hazard ratio (HR), 95% confidence interval (CI).

^a^ Median [interquartile range]

In the fully adjusted model (Model 3b), individuals in the highest quintile of MK-4 intake had 12% higher rate of dementia, compared with those in the lowest quintile (HR_Q5vsQ1_ [95%CI]: 1.12 [1.03–1.23], Supplementary Table 2). MK-9 was not associated with dementia incidence (HR_Q5vsQ1_ [95%CI]: 1.05 [0.96–1.14]; Model 3b, Supplementary Table 2).

Comparable trends were seen when the cumulative incidence of dementia by quintiles of vitamin K exposures was plotted using the Aalen-Johansen estimator, accounting for death as a competing risk (Supplementary Fig. 3).

Interaction analyses showed no evidence of effect modification by sex (K1×sex *p* = 0.84; K2×sex *p* = 0.75), BMI (K1×BMI *p* = 0.70; K2×BMI *p* = 0.77), smoking status (K1×smoke *p* = 0.40; K2×smoke *p* = 0.32), or prevalent diabetes (K1×diabetes *p* = 0.57; K2×diabetes *p* = 0.43).

### Sensitivity analyses

Additional adjustment for total energy intake in Model 2 did not materially alter the interpretation of the findings. Vitamin K1 intake was inversely associated with dementia incidence (HRQ_5vsQ1_ [95%CI]: 0.83 [0.75–0.91]), and the direction of association for vitamin K2 remained consistent, though the magnitude attenuated (HRQ_5vsQ1_ [95%CI]: 1.05 [0.95–1.17]; Supplementary Table 3). Further adjusting the analysis for baseline comorbidities and medication use did not materially change the results from primary analyses (K1 HR_Q5vsQ1_ [95%CI]: 0.85 [0.78–0.93], Model 3a.i; K2 HR_Q5vsQ1_ [95%CI]: 1.13 [1.04–1.24], Model 3b.i; Supplementary Table 4). After excluding participants with potentially implausible energy intakes at baseline (*n* = 972), results remained comparable with the primary analyses for both vitamin K1 (HR_Q5vsQ1_ [95%CI]: 0.84 [0.77–0.92], Model 3a) and K2 (HR_Q5vsQ1_ [95%CI]: 1.12 [1.03–1.23], Model 3b) (Supplementary Table 5). Similarly, associations did not change after excluding participants with dementia diagnosis within the first five years of follow-up (excluded participants *n* = 2,843) (vitamin K1 HR_Q5vsQ1_ [95%CI]: 0.86 [0.78–0.94], Model 3a; vitamin K2 HR_Q5vsQ1_ [95%CI]: 1.14 [1.04–1.25], Model 3b; Supplementary Table 6). When the follow-up period was restricted to 10 years, the inverse association between vitamin K1 intake and dementia incidence became stronger (HR_Q5vsQ1_ [95%CI]: 0.69 [0.51–0.93]; Model 3a, Supplementary Table 7), while vitamin K2 intakes were no longer associated with a higher rate of dementia (HR_Q5vsQ1_ [95%CI]: 0.99 [0.73–1.34]; Model 3b, Supplementary Table 7). After additional adjustment for processed meat intake, the positive association between vitamin K2 intake and dementia incidence was slightly attenuated albeit in the same direction and remained borderline (HR_Q5vsQ1_ [95%CI]: 1.10 [1.00–1.21], Model 3b; data not shown).

## Discussion

In this large, prospective cohort of Danish adults, we observed that higher dietary intakes of vitamin K1 were inversely associated with incident dementia over ~ 25 years of follow-up, with the association appearing to plateau at approximately 90 µg/day (Q3). Specifically, compared to those with the lowest intake, participants with the highest vitamin K1 intakes had a 14% lower rate of incident dementia. When analyses were restricted to 10 years of follow-up or to early-onset dementia cases, the association strengthened to 31% and 38% lower rates, respectively. In contrast, higher total dietary vitamin K2 intakes were associated with a 14% higher rate of incident dementia; this appeared to be driven primarily by MK-4. This relationship was attenuated in analyses restricted to 10 years of follow-up or when considering early-onset dementia. There was no evidence of effect modification by sex, BMI, smoking status, or diabetes status based on interaction analyses.

The inverse association between vitamin K1 intake and dementia risk in this study aligns with previous cross-sectional evidence [13–15, 17, 45, 46]. A systematic review of seven studies (six cross-sectional and one prospective) concluded that lower dietary or circulating concentrations of vitamin K1 were consistently associated with poorer cognitive performance in adults aged ≥ 65 years [46]. Cross-sectional studies have reported higher vitamin K1 intakes among those with higher Mini Mental State Examination (MMSE) scores, with intakes > 121 µg/day associated with fourfold greater odds of having normal cognitive function (OR [95%CI] 4.03 [1.19–13.65]) [14, 15]. However, the use of MMSE, a broad screening tool with limited sensitivity to subtle cognitive deficits, restricts the precision of cognitive function assessment in these studies [47]. Additionally, lower vitamin K1 intake was observed in those with severe subjective memory complaints [45] and in early-stage Alzheimer’s disease patients compared to cognitively healthy controls [13]. Nevertheless, these cross-sectional findings should be interpreted with caution, particularly in the context of dementia which can influence dietary habits/recall. Evidence from vitamin K biomarker studies also suggests poorer vitamin K status, indicated by elevated undercarboxylated osteocalcin, was associated with 65% higher odds of cognitive impairment (OR [95%CI]: 1.65 [1.06–2.59]) among 800 older Japanese adults [17].

While no longitudinal study has examined the association between vitamin K1 intake and incident dementia, prospective evidence on cognitive decline generally supports our findings. In the PREDIMED-Plus cohort (*n* = 5,533, 48.1% female), older adults (mean age 65.1 ± 4.9 years) with metabolic syndrome who increased their vitamin K1 intake by 194 µg/day over two years had 47% lower odds of cognitive decline compared to those who decreased their intake by 98 µg/day (OR [95%CI]: 0.53 [0.35–0.79]) [18]. However, interpretation of these results is complicated due to the absence of reported absolute intakes. Alternatively, the Longitudinal Aging Study Amsterdam (*n* = 599, mean age 59.9 ± 3.0 years, 54.2% female), found no association between desphospho-uncarboxylated MGP (dp-ucMGP) and cognitive decline, which was assessed using a comprehensive battery of tests evaluating information processing speed, episodic memory, and fluid intelligence over six years [19]. Nevertheless, the young age of this cohort limits generalisability of findings. Given that MGP is a VKDP primarily involved in vascular calcification [10], and the close link between vascular and cognitive health [48], this lack of association warrants further investigation.

Collectively, these findings support a potential role for vitamin K1 in cognitive health, which our study extends to long-term dementia outcomes. The observed threshold of benefit in this study is noteworthy, as participants in Q2 consumed amounts closer to current EFSA recommendations of ~ 1 µg/kg/day (70 µg/day) [49], whereas those who increased their consumption towards 90 µg/day appeared to derive the greatest benefit. This suggests that modest increases in vitamin K1 intake above current recommendations may confer additional advantages in the context of dementia. However, these intake estimates are based on self-reported dietary data and may over or underestimate actual intake. Moreover, foods rich in vitamin K1 also contain other nutrients and bioactive compounds that could contribute to the observed associations, suggesting that these relationships could reflect the combined influence of various dietary components and can be a marker of generally healthier dietary pattern rather than the isolated effect of vitamin K1. Pharmacological evidence further suggests a potential role for vitamin K in brain health. Warfarin, a vitamin K antagonist, has been associated with higher dementia risk, whereas DOACs, which do not interfere with vitamin K metabolism, are linked to lower risk [11]. While these associations may be confounded by indication, as patients prescribed warfarin vs. DOACs differ in unmeasured clinical characteristics that independently influence dementia risk [11], they raise the possibility that vitamin K contributes to maintaining cerebrovascular and neuronal health.

Several biological mechanisms may explain the role of vitamin K in cognitive health, with evidence suggesting that vitamin K1 and K2 may have distinct tissue distributions and biological roles [20]. Both vitamin K1 and vitamin K2 participate in sphingolipid metabolism, which is essential for neuronal membrane structure, myelin maintenance, and cellular signalling in the brain [4, 20, 46], pathways disrupted in Alzheimer’s disease [50]. MK-4 is the predominant form of vitamin K in brain tissue, where it can be derived through both direct dietary intake of K2 and conversion of K1 in tissues [4, 20]. Preclinical studies suggest that MK-4 regulates neuronal survival and inflammation through activating VKDPs such as Gas6 and Protein S which are expressed in the central nervous system. These proteins regulate microglial activity (immune cells in the nervous system) and reduce apoptosis induced by β-amyloid [4, 20]. Additionally, vitamin K2 may support cerebrovascular health by inhibiting vascular calcification, a contributor to cognitive decline, by activating MGP [10, 20]. However, these proposed neuroprotective mechanisms are derived primarily from in vitro and animal studies using isolated vitamin K2 compounds [4] and may not translate directly to the context of dietary vitamin K2 intake, which is consumed within a complex food matrix and accompanied with other nutrients and dietary behaviours.

In the present study, dietary vitamin K2 intake was not associated with lower dementia risk. Higher total vitamin K2 intake, primarily driven by MK-4, was associated with a 14% higher risk of dementia. This contrasts with findings from the Rush Memory and Aging Project (*n* = 325 decedents, mean age 92 ± 6, 75% female), where higher post-mortem MK-4 concentrations in brain tissue were linked to 17–20% lower odds of dementia and mild cognitive impairment before death, respectively [51]. Oral vitamin K2 administration in animal models also showed improved memory and cognitive performance [52, 53]. Contrastingly, in a study of 48 centenarians (aged ≥ 98 years, 89.6% female), cerebral MK-4 concentrations did not differ between individuals with and without dementia [54]. Additionally, no associations were observed between brain MK-4 levels and premortem cognitive function, whereas higher serum vitamin K1 was linked to better cognitive performance [54]. Collectively, the existing literature does not support a detrimental effect of vitamin K2 on cognitive health, suggesting that the positive association observed in the present study is more likely attributable to residual confounding by dietary patterns and food sources associated with vitamin K2 intake than to a direct effect of the nutrient itself. These inconsistencies nonetheless highlight the need for further research into the relationship between different forms of vitamin K, cognitive health and dementia.

To our knowledge, our research represents one of the earliest studies to examine dietary vitamin K2 intake in relation to dementia risk in a longitudinal population-based study. This distinction is important because unlike preclinical studies using isolated compounds, dietary vitamin K2 intake is closely linked to specific food sources that may independently influence dementia risk. In our cohort, individuals in the highest quintile of vitamin K2 intake consumed nearly twice as much red meat, processed meat, and dairy products, as those in the lowest quintile, with nearly 62% of vitamin K2 derived from these sources. They also had higher energy intake, a generally less healthy dietary pattern, and higher smoking intensity. Correlated dietary and lifestyle factors may have confounded the observed positive association with dementia risk, supported by the absence of a vitamin K2-dementia association among never smokers (exploratory analysis; not shown) and slight attenuation of the association after further adjustment for processed meat (a marker of less healthy dietary pattern [43]). This underscores the importance of considering food sources, dietary patterns, and lifestyle when evaluating dementia risk. Although we used regression models to mitigate such confounding effects, some residual and unmeasured confounding could still bias our estimates. Furthermore, as absorption and transportation of vitamin K in the body is highly dependent of triglycerides, this should be explored by future work [55].

This study has several strengths. It is the largest prospective study to examine dietary vitamin K1 and K2 and incident dementia, with up to 28 years of follow-up. This in combination with limited loss to follow-up allowed for substantial incident dementia case accumulation and good statistical power. The use of validated national health registries ensures complete follow-up. Additionally, the relatively young baseline age (median 56 years) enabled capture of mid-life dietary habits and risk factors (obesity, CVD, diabetes, CKD, physical inactivity, and smoking), which are particularly relevant given the role of mid-life risk factors in dementia development [56]. Detailed demographics, lifestyle, and dietary data allowed comprehensive confounder adjustment. However, several limitations should be considered.

As an observational study, residual or unmeasured confounding cannot be excluded and causality cannot be inferred. Given that vitamin K1 intake correlates with healthy diet and lifestyle, some residual confounding may remain despite multivariable adjustment. Although 28 years of follow-up captured the long pre-clinical phase of dementia, the single time point assessment of diet over this duration may have led to exposure misclassification, as dietary habits can change over time. Furthermore, changes in other lifestyle factors and health conditions may have introduced additional unmeasured confounding. Such non-differential misclassification and unmeasured confounding generally bias associations toward the null. The stronger associations observed for vitamin K1 when follow-up was restricted to 10 years are consistent with this, whereas the vitamin K2 association attenuated with shorter follow-up. These findings should be interpreted with caution given the smaller number of events in the restricted follow-up analyses. Future studies with repeated dietary assessments are needed. In addition, while MK-4 contributed the largest proportion of total vitamin K2 intake in this cohort, it was available for a greater proportion of food items than other menaquinones, which are less consistently available in existing food composition databases, and their contributions may therefore be underestimated. Dementia diagnoses were based on national health registry data. However, without baseline or follow-up cognitive assessments, undiagnosed cognitive impairment would not have been identified, and dementia incidence may have been underestimated if individuals neither received anti-dementia medication nor had a formal diagnosis recorded. Additionally, this study outcome was all-cause dementia, which comprises various subtypes with differing underlying pathophysiology, including Alzheimer’s disease and vascular dementia. Given that vitamin K has been implicated in multiple biological pathways, its association may differ across dementia subtypes. However, due to the use of registry-based data, we were not able to reliably distinguish between dementia subtypes. As such, the observed associations may vary across specific dementia types and should be interpreted accordingly. As the cohort consisted predominantly of White Danish adults, generalisability to more diverse populations may be limited. Finally, the early-onset dementia analysis should be interpreted with caution due to potential selection bias. Individuals recruited at older ages who survived and met eligibility criteria likely represent a healthier subset of their age group, potentially leading to underestimation of early-onset dementia risk.

## Conclusion

In conclusion this large prospective study demonstrates that higher dietary vitamin K1 intake is associated with a lower risk of developing dementia, whereas higher vitamin K2 intake, particularly MK-4, was associated with higher risk. These contrasting associations likely reflect differences in dietary sources and accompanying dietary patterns. It is possible that the observed associations for both vitamin K1 and vitamin K2 may therefore reflect residual confounding by dietary pattern not fully captured by several dietary covariates included in our models, rather than a direct causal effect of the nutrients themselves. Collectively, these findings highlight the need to differentiate between vitamin K forms and their food sources when evaluating the relationship between diet and dementia. From a public health perspective, these findings reinforce the importance of promoting vitamin K1-rich vegetables, including green leafy vegetables (such as spinach, lettuce, etc.,) and cruciferous vegetables (such as cabbages, broccoli, etc.,) as part of broader dietary patterns supportive of cognitive health. Alternatively, the observed association for vitamin K2 should be interpreted with caution and may reflect residual dietary patterns associated with higher consumption of vitamin K2-rich animal-based foods and warrants further investigation.

## Supplementary Information

Below is the link to the electronic supplementary material.

Supplementary Material 1

## Data Availability

The datasets from the Danish Diet Cancer and Health cohort presented in this article are not readily available due to the sensitive nature of the data collected for this study. Requests to access the dataset from qualified researchers trained in human subject confidentiality protocols may be sent to the Diet Cancer and Health Steering Committee at the Danish Cancer Institute (dch@cancer.dk).
