# Supplementary material for: Associations between dietary vitamin K1 and K2 intake and incident dementia: findings from the Danish Diet, Cancer, and Health cohort
Source: Eur J Nutr. 2026 Aug 6;65(6):229. doi: 10.1007/s00394-026-04081-w (PMC13447346; doi:10.1007/s00394-026-04081-w)
Supplement: Supplementary file 1 [file 394_2026_4081_MOESM1_ESM.docx]

**Associations between dietary vitamin K1 and K2 intake and incident dementia: findings from the Danish Diet, Cancer, and Health cohort.**

Negar Ghasemifard^1^, Marc Sim^1,2,3^, Pratik Pokharel^1,4α^, Chengfeng Li^1^, Allan Linneberg^5,6^, Anne Ahrendt Bjerregaard^5^, Anja Olsen^4,7^, Christina C. Dahm^7^, Simone Radavelli-Bagatini^1^, Simon M. Laws^8,9^, Josh R. Lewis^1,2^, Jonathan M. Hodgson^1^, Nicola P. Bondonno^1,4α^

^1^ Nutrition and Health Innovation Research Institute, School of Medical and Health Sciences, Edith Cowan University, Perth, Western Australia, Australia.

^2^ Medical School, University of Western Australia, Crawley, WA, Australia.

^3^ Royal Perth Hospital Research Foundation, Perth, WA, Australia.

^4^ The Danish Cancer Institute, Copenhagen, Denmark.

^5^ Centre for Clinical Research and Prevention, Copenhagen University Hospital, Bispebjerg and Frederiksberg, Frederiksberg, Denmark.

^6^ Department of Clinical Medicine, The Faculty of Health and Medical Sciences, University of Copenhagen, Copenhagen, Denmark.

^7^ Department of Public Health, Aarhus University, Aarhus, Denmark.

^8^ Centre for Precision Health, Edith Cowan University, Joondalup, WA, Australia.

^9^ Collaborative Genomics and Translation Group, School of Medical and Health Sciences, Edith Cowan University, Joondalup, WA, Australia.

^α^ Corresponding author at: Nutrition & Health Innovation Research Institute, School of Medical and Health Sciences, Edith Cowan University, Level 3 Royal Perth Hospital Medical Research Foundation Building, Rear of 50 Murray Street, Perth, WA, 6000, Australia.

E-mail address: [n.bondonno@ecu.edu.au](mailto:n.bondonno@ecu.edu.au) / [p.pokharel@ecu.edu.au](mailto:p.pokharel@ecu.edu.au)

**Table of Contents**

**Supplementary Figure 1**	**3**

**Supplementary Table 1**	**4-6**

**Supplementary Figure 2**	**7**

**Supplementary Table 2**	**8**

**Supplementary Figure 3**	**9**

**Supplementary Table 3**	**10**

**Supplementary Table 4**	**11**

**Supplementary Table 5**	**12**

**Supplementary Table 6**	**13**

**Supplementary Table 7**	**14**

**Supplementary Figure 1:** CONSORT flow diagram of the Danish Diet Cancer and Health study participants included in present study.

Abbreviations: food frequency questionnaire (FFQ), vitamin K antagonist (VKA).

| **Supplementary Table 1:** Covariate coding and categorisation information for the Danish Diet Cancer and Health study | | |
| --- | --- | --- |
| **Variables** | **Categorisation/units** | **Variable description** |
| **Outcome** |  |  |
| Dementia | No; Yes | ICD-10 diagnosis [codes: F00, F01, F02, F03, G30, G31.0B, G31.8, G31.8E, G31.9] in the Danish National Patient Register, or the prescription of a dementia-related medication [ATC code: N06D] in the Danish National Prescription Registry. |
| **Demographics** |  |  |
| Age | Years | Age at recruitment |
| Sex | Female; Male | Sex |
| BMI | Underweight (<18.5 kg/m^2^); Healthy weight (18.5-24.99 kg/m^2^); Overweight (25-29.99 kg/m^2^); Obese (30-30.99 kg/m^2^); Morbidly obese (≥40 kg/m^2^) | Height and weight were objectively measured at baseline and body mass index (BMI) was calculated in kg/m². |
| **Socioeconomic status** |  |  |
| Education | Educational levels were categorized as follows: "Short" denoted mandatory schooling, limited to seven years for those born before January 1, 1958, and nine years for those born after. "Medium" encompassed secondary and vocational education, corresponding to 10-12 years of schooling. "Higher" encompassed various levels of tertiary education, typically exceeding 12 years of education. | Obtained from the Education Registry |
| **Diet and Lifestyle** |  |  |
| Energy intake | Kilo calorie/day | Calculated from the food frequency questionnaire |
| Individual food and beverage items | Grams or millilitre/day | Calculated from the food frequency questionnaire |
| Alcohol intake | Grams/day | Calculated from the food frequency questionnaire |
| Smoking status | Current/former/never | Self-reported smoking status |
| Smoking packyears | packyears | Calculated from the number of cigarettes smoked per day divided by 20 and multiplied by the number of years smoked |
| Physical activity | METs hr/week | Self-reported daily physical activity. |
| **History of disease and medication** |  |  |
| Prevalent diabetes | No; Yes | A diagnosis of either type 1 diabetes or type 2 diabetes in the RUKS registry before or on baseline date. The RUKS register defines type 1 diabetes as no prescription purchases of medication for type 2 diabetes only (see ATC codes below) in combination with at least two purchases on different dates of medicines approved for type 1 diabetes (ATC codes: A10A except when in combination with A10AE54, and A10AE56). The RUKS register defines type 2 diabetes as a validated diagnostic record of a hospital contact with a type 2 diabetes diagnosis [ICD-10 codes: E11*] recorded in The Danish National Patient Registry or at least two prescription purchases (on different dates) of medication for type 2 diabetes but not type 1 diabetes [ATC codes: A10AE54, A10AE56 and A10B excl. A10BJ, A10BK01, A10BK03, A10BX16) recorded in the Danish National Prescription Registry. |
| Prevalent CVD | No; Yes | Defined as either of the following before or on baseline date:  - Self-reported ischemic heart disease OR  - Diagnosis of ischemic heart disease (primary or secondary diagnosis recorded during hospital admission, outpatient clinic visit, or emergency room visit with an ICD-8 diagnosis code of 410-414 or an ICD-10 diagnosis code of I20-I25) OR  - Self reported stroke OR  - Diagnosis of ischemic stroke (primary or secondary diagnosis recorded during hospital admission, outpatient clinic visit, or emergency room visit with an ICD-8 diagnosis code of 433-434 or an ICD-10 diagnosis code of I63) OR  - Diagnosis of haemorrhagic stroke (primary or secondary diagnosis recorded during hospital admission, outpatient clinic visit, or emergency room visit with an ICD-8 diagnosis code of 430-432 or an ICD-10 diagnosis code of I60-I62) OR  - Diagnosis of peripheral artery disease (primary or secondary diagnosis recorded during hospital admission, outpatient clinic visit, or emergency room visit with an ICD-8 diagnosis code of 440-444 or an ICD-10 diagnosis code of I70-I74) |
| Prevalent CKD | No; Yes | A primary or secondary diagnosis recorded during hospital admission, outpatient clinic visit, or emergency room visit with an ICD-8 diagnosis code of 580-584 or an ICD-10 diagnosis code of E102, E112, E122, E132, E142, I12, I13, N02-N06, N083, N11, N12, N158, N159, N162, N164, N165, N168, N18, N19, N26, Z49, Z992 OR an SKS code of BJFD20-BJFD27, KKAS00, KKAS10, KKAS20 prior to baseline. |
| Use of cholesterol-lowering medication at baseline | No; Yes | A claimed prescription of cholesterol-lowering medication claimed within 180 days prior to study enrolment [ATC code: C10AA]. |
| Use of antihypertensives at baseline | No; Yes | A claimed prescription of two or more antihypertensive medications claimed within 180 days prior to study enrolment [ATC codes: C02A, C02B, C02C, C02DA, C02DB, C02DD, C02DG, C02L, C03A, C03B, C03C, C03D, C03E, C03EA, C03EB, C03X, C07A, C07B, C07C, C07D, C07F, C08, C08G, C09AA, C09BA, C09BB, C09CA, C09DA, C09DB, C09XA02, C09XA52]. |
| Vitamin K-antagonist medication at baseline | No; Yes | A claimed prescription of a vitamin K antagonist claimed within 180 days prior to study enrolment [ATC code: B01AA]. |
| Vitamin K-antagonist medication after baseline | No; Yes | A claimed prescription of a vitamin K antagonist claimed after enrolment [ATC code: B01AA]. |
| Abbreviations: Body Mass Index (BMI); Metabolic Equivalent (MET); the Register for Selected Chronic Diseases (RUKS); Cardiovascular Disease (CVD); Chronic Kidney Disease (CKD). | | |

**Supplementary Figure 2:** Food group contributions to total vitamin K1 and vitamin K2 intake. Percentage contribution of major food groups to total dietary intake of a) vitamin K1 and b) vitamin K2 among study participants.

| **Supplementary Table 2:** Hazard ratios for incident dementia by quintiles of vitamin K2 vitamers intake. | | | | | |
| --- | --- | --- | --- | --- | --- |
|  | Vitamin K intake quintiles | | | | |
|  | Quintile 1  n=10,994 | Quintile 2  n=10,994 | Quintile 3  n=10,994 | Quintile 4  n=10,993 | Quintile 5  n=10,993 |
| MK-4 | | | | | |
| Intake, µg PKeq/day ^a^ | 19.8 [16.8 – 22.0] | 27.2 [25.6 – 28.8] | 33.6 [32.0 – 35.2] | 41.0 [38.9 – 43.4] | 54.9 [49.8 – 63.8] |
| Number of events (%) | 958 (8.7) | 1022 (9.3) | 940 (8.5) | 972 (8.8) | 910 (8.3) |
| HR (95%CI) | | | | | |
| Model 1 | Ref. | 1.03 (0.98-1.09) | 1.04 (0.98-1.11) | 1.05 (0.98-1.13) | **1.08 (1.00-1.17)** |
| Model 2 | Ref. | 1.05 (0.99-1.11) | 1.06 (0.99-1.13) | 1.05 (0.98-1.13) | 1.06 (0.98-1.15) |
| Model 3b | Ref. | **1.08 (1.02-1.14)** | **1.10 (1.03-1.18)** | **1.10 (1.02-1.19)** | **1.12 (1.03-1.23)** |
| MK-9 | | | | | |
| Intake, µg PKeq/day ^a^ | 2.25 [1.45 – 2.95] | 4.30 [3.96 – 4.70] | 6.73 [5.86 – 8.03] | 9.77 [9.26 – 10.49] | 15.47 [13.36 – 18.35] |
| Number of events (%) | 958 (8.7) | 905 (8.2) | 931 (8.5) | 973 (8.8) | 1035 (9.4) |
| HR (95%CI) | | | | | |
| Model 1 | Ref. | **0.92 (0.87-0.97)** | **0.90 (0.84-0.97)** | 0.94 (0.88-1.01) | 0.98 (0.91-1.06) |
| Model 2 | Ref. | **0.94 (0.88-0.99)** | 0.93 (0.86-1.00) | 0.97 (0.91-1.05) | 1.02 (0.94-1.10) |
| Model 3b | Ref. | 0.95 (0.90-1.01) | 0.95 (0.88-1.03) | 1.00 (0.93-1.08) | 1.05 (0.96-1.14) |
| Hazard ratios (95% confidence intervals) for dementia incident over 28 years of follow-up, obtained from restricted cubic splines based on Cox proportional hazards models, comparing the median intake in quintiles 2 – 5, to the median intake in quintile 1. Bolded indicates p <0.05 compared to Q1. Model 1 adjusted for sex and age; model 2 adjusted for sex, age, body mass index, physical activity, education, smoking status, smoking pack per year, living situation, and alcohol intake; model 3b adjusted for all covariates in model 2 plus intake of vegetables, fruits, wholegrains, refined grains, tea, coffee and sugar-sweetened soft drink.  ^a^ Median [interquartile range]  Abbreviations: Phylloquinone equivalent (PKeq), Hazard ratio (HR), 95% confidence interval (CI). | | | | | |

**Supplementary Figure 3:** unadjusted cumulative incidence of dementia accounting for death as a competing risk using the Aalen-Johansen estimator, across quintiles of a) vitamin K1 and b) vitamin K2

| **Supplementary Table 3:** Hazard ratios for incident dementia by quintiles of vitamin K intake including the additional adjustment for energy intake to Model 2. | | | | | |
| --- | --- | --- | --- | --- | --- |
|  | Vitamin K intake quintiles | | | | |
|  | Quintile 1  n=10,994 | Quintile 2  n=10,994 | Quintile 3  n=10,994 | Quintile 4  n=10,993 | Quintile 5  n=10,993 |
| Vitamin K1 | | | | | |
| Intake, µg/day ^a^ | 48.8 [40.6 – 55.0] | 70.2 [65.5 – 74.8] | 88.1 [83.6 – 93.0] | 108.8 [103.0 – 115.3] | 146.2 [132.5 – 168.4] |
| Number of events (%) | 1011 (9.2) | 957 (8.7) | 942 (8.6) | 977 (8.9) | 915 (8.3) |
| HR (95%CI) | | | | | |
| Model 2 + energy intake | Ref. | **0.91 (0.86-0.96)** | **0.87 (0.81-0.93)** | **0.85 (0.78-0.92)** | **0.83 (0.75-0.91)** |
| Vitamin K2 | | | | | |
| Intake, µg PKeq/day ^a^ | 29.1 [25.0 – 32.4] | 39.5 [37.3 – 41.6] | 47.8 [45.8 – 50.0] | 57.3 [54.6 – 60.3] | 74.4 [68.3 – 84.5] |
| Number of events (%) | 943 (8.6) | 973 (8.9) | 966 (8.8) | 941 (8.6) | 979 (8.9) |
| HR (95%CI) | | | | | |
| Model 2 + energy intake | Ref. | 1.04 (0.98-1.11) | 1.05 (0.97-1.13) | 1.04 (0.95-1.14) | 1.05 (0.95-1.17) |
| MK-4 | | | | | |
| Intake, µg PKeq/day ^a^ | 19.8 [16.8 – 22.0] | 27.2 [25.6 – 28.8] | 33.6 [32.0 – 35.2] | 41.0 [38.9 – 43.4] | 54.9 [49.8 – 63.8] |
| Number of events (%) | 958 (8.7) | 1022 (9.3) | 940 (8.5) | 972 (8.8) | 910 (8.3) |
| HR (95%CI) | | | | | |
| Model 2 + energy intake | Ref. | 1.06 (1.00-1.13) | 1.07 (0.99-1.15) | 1.04 (0.96-1.14) | 1.04 (0.94-1.15) |
| MK-9 | | | | | |
| Intake, µg PKeq/day ^a^ | 2.25 [1.45 – 2.95] | 4.30 [3.96 – 4.70] | 6.73 [5.86 – 8.03] | 9.77 [9.26 – 10.49] | 15.47 [13.36 – 18.35] |
| Number of events (%) | 958 (8.7) | 905 (8.2) | 931 (8.5) | 973 (8.8) | 1035 (9.4) |
| HR (95%CI) | | | | | |
| Model 2 + energy intake | Ref. | 0.93 (0.88-0.99) | 0.92 (0.86-0.99) | 0.96 (0.89-1.04) | 1.00 (0.92-1.09) |
| Hazard ratios (95% confidence intervals) for dementia incidence over 28 years of follow-up, obtained from restricted cubic splines based on Cox proportional hazards models, comparing the median intake in quintiles 2 – 5, to the median intake in quintile 1. Bolded indicates p <0.05 compared to Q1. model 2 adjusted for sex, age, body mass index, physical activity, education, smoking status, smoking pack per year, living situation, and alcohol intake.  ^a^ Median [interquartile range]  Abbreviations: Phylloquinone equivalent (PKeq), Hazard ratio (HR), 95% confidence interval (CI). | | | | | |

| **Supplementary Table 4:** Hazard ratios for incident dementia by quintiles of vitamin K intake including the additional adjustment for baseline comorbidities and medication use. | | | | | |
| --- | --- | --- | --- | --- | --- |
|  | Vitamin K intake quintiles | | | | |
|  | Quintile 1  n=10,994 | Quintile 2  n=10,994 | Quintile 3  n=10,994 | Quintile 4  n=10,993 | Quintile 5  n=10,993 |
| Vitamin K1 | | | | | |
| Intake, µg/day ^a^ | 48.8 [40.6 – 55.0] | 70.2 [65.5 – 74.8] | 88.1 [83.6 – 93.0] | 108.8 [103.0 – 115.3] | 146.2 [132.5 – 168.4] |
| Number of events (%) | 1011 (9.2) | 957 (8.7) | 942 (8.6) | 977 (8.9) | 915 (8.3) |
| HR (95%CI) | | | | | |
| Model 3a.i | Ref. | **0.91 (0.86-0.97)** | **0.88 (0.82-0.94)** | **0.87 (0.80-0.94)** | **0.85 (0.78-0.93)** |
| Vitamin K2 | | | | | |
| Intake, µg PKeq/day ^a^ | 29.1 [25.0 – 32.4] | 39.5 [37.3 – 41.6] | 47.8 [45.8 – 50.0] | 57.3 [54.6 – 60.3] | 74.4 [68.3 – 84.5] |
| Number of events (%) | 943 (8.6) | 973 (8.9) | 966 (8.8) | 941 (8.6) | 979 (8.9) |
| HR (95%CI) | | | | | |
| Model 3b.i | Ref. | 1.05 (1.00-1.11) | **1.08 (1.01-1.15)** | **1.10 (1.02-1.19)** | **1.13 (1.04-1.24)** |
| Hazard ratios (95% confidence intervals) for dementia incidence over 28 years of follow-up, obtained from restricted cubic splines based on Cox proportional hazards models, comparing the median intake in quintiles 2 – 5, to the median intake in quintile 1. Bolded indicates p <0.05 compared to Q1. Model 3a.i adjusted for sex, age, body mass index, physical activity, education, smoking status, smoking pack per year, living situation, alcohol intake, intakes of red meat, poultry, processed meat, fish, dairy, refined grains, tea, coffee, sugar-sweetened soft drink, prevalent diabetes, prevalent cardiovascular disease, prevalent chronic kidney disease, and the use of antihypertensive medications, and statin. model 3b.i adjusted for sex, age, body mass index, physical activity, education, smoking status, smoking pack per year, living situation, alcohol intake, intake of vegetables, fruits, wholegrains, refined grains, tea, coffee and sugar-sweetened soft drink, prevalent diabetes, prevalent cardiovascular disease, prevalent chronic kidney disease, and the use of antihypertensive medications, and statin.  ^a^ Median [interquartile range]  Abbreviations: Phylloquinone equivalent (PKeq), Hazard ratio (HR), 95% confidence interval (CI). | | | | | |

| **Supplementary Table 5:** Hazard ratios for incident dementia by quintiles of vitamin K intake excluding participants with implausible energy intake at baseline. | | | | | |
| --- | --- | --- | --- | --- | --- |
|  | Vitamin K intake quintiles | | | | |
|  | Quintile 1  n=10,800 | Quintile 2  n=10,799 | Quintile 3  n=10,799 | Quintile 4  n=10,799 | Quintile 5  n=10,799 |
| Vitamin K1 | | | | | |
| Intake, µg/day ^a^ | 48.6 [40.5 – 54.7] | 69.9 [65.1 – 74.3] | 87.4 [83.0 – 92.2] | 107.7 [102.2 – 114.0] | 144.1 [130.8 – 165.3] |
| Number of events (%) | 994 | 952 | 909 | 951 | 891 |
| HR (95%CI) | | | | | |
| Model 1 | Ref. | **0.90 (0.85-0.95)** | **0.86 (0.81-0.92)** | **0.85 (0.79-0.91)** | **0.83 (0.77-0.90)** |
| Model 2 | Ref. | **0.92 (0.87-0.98)** | **0.90 (0.84-0.96)** | **0.90 (0.83-0.97)** | **0.88 (0.81-0.95)** |
| Model 3a | Ref. | **0.91 (0.86-0.97)** | **0.89 (0.83-0.95)** | **0.87 (0.81-0.95)** | **0.84 (0.77-0.92)** |
| Vitamin K2 | | | | | |
| Intake, µg PKeq/day ^a^ | 29.0 [24.9 – 32.2] | 39.3 [37.1 – 41.4] | 47.4 [45.4 – 49.6] | 56.7 [54.1 – 59.6] | 72.7 [67.1 – 81.8] |
| Number of events (%) | 925 | 960 | 947 | 922 | 943 |
| HR (95%CI) | | | | | |
| Model 1 | Ref. | 1.01 (0.95-1.06) | 1.02 (0.95-1.08) | 1.03 (0.96-1.11) | 1.05 (0.97-1.14) |
| Model 2 | Ref. | 1.02 (0.97-1.08) | 1.04 (0.97-1.10) | 1.04 (0.97-1.12) | 1.05 (0.97-1.14) |
| Model 3b | Ref. | **1.05 (1.00-1.12)** | **1.08 (1.01-1.16)** | **1.10 (1.02-1.20)** | **1.12 (1.03-1.23)** |
| Hazard ratios (95% confidence intervals) for dementia incident over 28 years of follow-up excluding participants with implausible energy intake (n= 972; <800 or >4,200 kcal/day for men and <500 or >3,500 kcal/day for women) at baseline. Hazard ratios obtained from restricted cubic splines based on Cox proportional hazards models, comparing the median intake in quintiles 2 – 5, to the median intake in quintile 1. Bolded indicates p <0.05 compared to Q1. Model 1 adjusted for sex and age; model 2 adjusted for sex, age, body mass index, physical activity, education, smoking status, smoking pack per year, living situation, and alcohol intake; model 3a adjusted for all covariates in model 2 plus intakes of red meat, poultry, processed meat, fish, dairy, refined grains, tea, coffee and sugar-sweetened soft drink; model 3b adjusted for all covariates in model 2 plus intake of vegetables, fruits, wholegrains, refined grains, tea, coffee and sugar-sweetened soft drink.  ^a^ Median [interquartile range]  Abbreviations: Phylloquinone equivalent (PKeq), Hazard ratio (HR), 95% confidence interval (CI). | | | | | |

| **Supplementary Table 6:** Hazard ratios for incident dementia by quintiles of vitamin K intake excluding participants with dementia diagnosis within 5 years from baseline. | | | | | |
| --- | --- | --- | --- | --- | --- |
|  | Vitamin K intake quintiles | | | | |
|  | Quintile 1  n=10,425 | Quintile 2  n=10,425 | Quintile 3  n=10,425 | Quintile 4  n=10,425 | Quintile 5  n=10,425 |
| Vitamin K1 | | | | | |
| Intake, µg/day ^a^ | 49.0 [40.9 – 55.3] | 70.5 [65.8 – 75.0] | 88.3 [83.8 – 93.1] | 108.9 [103.2 – 115.4] | 146.2 [132.5 – 168.5] |
| Number of events (%) | 990 | 937 | 917 | 952 | 888 |
| HR (95%CI) | | | | | |
| Model 1 | Ref. | **0.91 (0.86-0.96)** | **0.87 (0.82-0.93)** | **0.86 (0.80-0.93)** | **0.86 (0.79-0.93)** |
| Model 2 | Ref. | **0.93 (0.88-0.98)** | **0.91 (0.85-0.97)** | **0.91 (0.84-0.97)** | **0.90 (0.83-0.98)** |
| Model 3a | Ref. | **0.92 (0.87-0.97)** | **0.89 (0.83-0.95)** | **0.88 (0.81-0.95)** | **0.86 (0.78-0.94)** |
| Vitamin K2 | | | | | |
| Intake, µg PKeq/day ^a^ | 29.1 [24.9 – 32.3] | 39.5 [37.3 – 41.6] | 47.8 [45.7 – 50.0] | 57.2 [54.6 – 60.2] | 74.1 [68.1 – 84.2] |
| Number of events | 920 | 945 | 935 | 919 | 965 |
| HR (95%CI) | | | | | |
| Model 1 | Ref. | 1.00 (0.95-1.06) | 1.01 (0.95-1.08) | 1.04 (0.96-1.11) | **1.08 (1.00-1.17)** |
| Model 2 | Ref. | 1.02 (0.96-1.07) | 1.03 (097-1.10) | 1.04 (0.97-1.12) | 1.07 (0.99-1.16) |
| Model 3b | Ref. | 1.04 (0.99-1.10) | **1.07 (1.00-1.15)** | **1.10 (1.02-1.19)** | **1.14 (1.04-1.25)** |
| Hazard ratios (95% confidence intervals) for dementia incident over 28 years of follow-up excluding participants with dementia diagnosis within 5 years from baseline (excluded participants n= 2,843). Hazard ratios obtained from restricted cubic splines based on Cox proportional hazards models, comparing the median intake in quintiles 2 – 5, to the median intake in quintile 1. Bolded indicates p <0.05 compared to Q1. Model 1 adjusted for sex and age; model 2 adjusted for sex, age, body mass index, physical activity, education, smoking status, smoking pack per year, living situation, and alcohol intake; model 3a adjusted for all covariates in model 2 plus intakes of red meat, poultry, processed meat, fish, dairy, refined grains, tea, coffee and sugar-sweetened soft drink; model 3b adjusted for all covariates in model 2 plus intake of vegetables, fruits, wholegrains, refined grains, tea, coffee and sugar-sweetened soft drink.  ^a^ Median [interquartile range]  Abbreviations: Phylloquinone equivalent (PKeq), Hazard ratio (HR), 95% confidence interval (CI). | | | | | |

| **Supplementary Table 7:** Hazard ratios for incident dementia by quintiles of vitamin K intake restricting follow-up period to 10 years. | | | | | |
| --- | --- | --- | --- | --- | --- |
|  | Vitamin K intake quintiles | | | | |
|  | Quintile 1  n=10,994 | Quintile 2  n=10,994 | Quintile 3  n=10,994 | Quintile 4  n=10,993 | Quintile 5  n=10,993 |
| Vitamin K1 | | | | | |
| Intake, µg/day ^a^ | 48.8 [40.6 – 55.0] | 70.2 [65.5 – 74.8] | 88.1 [83.6 – 93.0] | 108.8 [103.0 – 115.3] | 146.2 [132.5 – 168.4] |
| Number of events (%) | 110 | 89 | 85 | 66 | 80 |
| HR (95%CI) | | | | | |
| Model 1 | Ref. | **0.79 (0.68-0.93)** | **0.72 (0.60-0.87)** | **0.70 (0.56-0.88)** | **0.68 (0.53-0.87)** |
| Model 2 | Ref. | 0.85 (0.72-1.00) | **0.80 (0.66-0.97)** | 0.79 (0.62-1.00) | 0.77 (0.59-1.01) |
| Model 3a | Ref. | 0.86 (0.72-1.02) | **0.79 (0.64-097)** | **0.75 (0.58-0.96)** | **0.69 (0.51-0.93)** |
| Vitamin K2 | | | | | |
| Intake, µg PKeq/day ^a^ | 29.1 [25.0 – 32.4] | 39.5 [37.3 – 41.6] | 47.8 [45.8 – 50.0] | 57.3 [54.6 – 60.3] | 74.4 [68.3 – 84.5] |
| Number of events (%) | 84 | 91 | 91 | 88 | 76 |
| HR (95%CI) | | | | | |
| Model 1 | Ref. | 1.14 (0.94-1.38) | 1.04 (0.84-1.30) | 0.87 (0.68-1.12) | 0.84 (0.64-1.10) |
| Model 2 | Ref. | 1.19 (0.98-1.44) | 1.10 (0.88-1.37) | 0.92 (0.72-1.19) | 0.88 (0.67-1.15) |
| Model 3b | Ref. | **1.26 (1.03-1.53)** | 1.20 (0.95-1.51) | 1.03 (0.79-1.34) | 0.99 (0.73-1.34) |
| Hazard ratios (95% confidence intervals) for dementia incidence over 10 years of follow-up, obtained from restricted cubic splines based on Cox proportional hazards models, comparing the median intake in quintiles 2 – 5, to the median intake in quintile 1. Bolded indicates p <0.05 compared to Q1. Model 1 adjusted for sex and age; model 2 adjusted for sex, age, body mass index, physical activity, education, smoking status, smoking pack per year, living situation, and alcohol intake; model 3a adjusted for all covariates in model 2 plus intakes of red meat, poultry, processed meat, fish, dairy, refined grains, tea, coffee and sugar-sweetened soft drink when vitamin K1 is the exposure of interest; model 3b adjusted for all covariates in model 2 plus intake of vegetables, fruits, wholegrains, refined grains, tea, coffee and sugar-sweetened soft drink when vitamin K2 is the exposure of interest.  ^a^ Median [interquartile range]  Abbreviations: Phylloquinone equivalent (PKeq), Hazard ratio (HR), 95% confidence interval (CI). | | | | | |
